# Real-Time Single-Cell Measurement and Kinetic Modeling of Daunorubicin Uptake in Multidrug-Resistant Leukemia Cells Using a Microfluidic Biochip

**DOI:** 10.3390/pathophysiology33020028

**Published:** 2026-04-21

**Authors:** Yuchun Chen, Megan Chiem, Nandini Joshi, Paul C. H. Li

**Affiliations:** Department of Chemistry, Simon Fraser University, Burnaby, BC V5A 1S6, Canada

**Keywords:** multidrug resistance, leukemia, daunorubicin, single-cell analysis, microfluidic biochip, drug uptake kinetics

## Abstract

**Background/Objectives**: Multidrug resistance (MDR) remains a major pathophysiological barrier to effective chemotherapy based on anthracyclines, including daunorubicin (DNR), in the treatment of leukemia. However, conventional population-level measurements of drug uptake do not resolve variability in uptake kinetics among individual leukemia cells, which may influence intracellular drug accumulation and therapeutic response. **Methods**: In this study, real-time DNR uptake was quantified at the single-cell level using a microfluidic biochip that enabled long-term cellular retention and continuous monitoring. Both wild-type drug-sensitive leukemia cells and a multidrug-resistant mutant overexpressing the P-glycoprotein (P-gp) efflux pump were examined. **Results**: Kinetic analysis revealed that DNR uptake in drug-sensitive cells was well described by a single dominant uptake process, whereas uptake in MDR cells required a model incorporating two kinetically distinct processes. In both cell populations, pronounced cell-to-cell variation was observed in uptake rates and intracellular drug retention, indicating substantial functional heterogeneity within phenotypically similar cells. This variability persisted following the treatment with an MDR inhibitor and obscured the differences between inhibitor-treated and untreated cells when the uptake was compared across different single cells. To overcome this limitation, a same-single-cell analysis (SASCA) approach was employed, enabling direct comparison of DNR uptake in the same individual cell before and after inhibitor exposure, thereby revealing enhanced intracellular DNR retention and accelerated uptake kinetics following inhibition. **Conclusions**: Together, these results demonstrate that real-time single-cell kinetic analysis reveals functionally relevant heterogeneity in multidrug-resistant leukemia cells and provides insight into the pathophysiology of MDR that cannot be obtained from population-averaged measurements.

## 1. Introduction

Multidrug resistance (MDR) in leukemia is characterized by impaired intracellular accumulation of chemotherapeutic agents [1,2], thereby reducing cytotoxic effectiveness. In anthracycline drugs such as daunorubicin (DNR), therapeutic outcome is dependent on drug exposure and equally contingent on the rate and extent of cellular uptake and retention [3,4]. These processes are inherently dynamic and can vary substantially between individual cells, even within the same cell population. Since DNR is intrinsically fluorescent [5,6], it provides a unique opportunity to directly monitor intracellular drug accumulation in real tme, enabling kinetic analysis of drug uptake behavior at the single-cell level using microfluidic platforms [7,8].

A key mediator of reduced intracellular drug accumulation in MDR leukemia cells is the activity of ATP-binding cassette transporters, particularly P-glycoprotein (P-gp), encoded by the MDR1 gene (ABCB1) [2,9]. MDR is commonly associated with the emergence of cross-resistance to structurally unrelated chemotherapeutic agents following prolonged drug exposure, a phenomenon closely linked to the altered drug transport kinetics. P-gp actively exports a broad range of chemotherapeutic agents, including DNR, thereby altering both the rate and extent of intracellular drug retention [1,5]. From a kinetic perspective, P-gp-mediated efflux introduces an additional transport process that can complicate the interpretation of drug uptake behavior [10]. Early kinetic studies of P-glycoprotein activity have similarly shown that drug accumulation reflects the balance between uptake and active efflux processes, and that modulation of P-gp function can significantly alter intracellular drug exposure [11]. In addition, clinical studies have evaluated P-gp inhibitors in combination with daunorubicin-based chemotherapy to enhance intracellular accumulation and improve treatment responses in acute leukemia [12,13]. As a result, intracellular drug accumulation in MDR cells may reflect the combined influence of passive uptake, active efflux, and intracellular redistribution, rather than a single dominant transport mechanism. Recent single-cell studies have shown that such heterogeneity in drug transport and MDR phenotypes can be resolved only at the level of individual cells and is frequently obscured in population-averaged measurements [14].

To characterize MDR-associated drug transport, previous studies have characterized the kinetics of DNR uptake and efflux in drug-sensitive and drug-resistant leukemia cells using population-level techniques, such as flow cytometry [15,16]. These approaches typically measure intracellular drug fluorescence at discrete time points across large numbers of cells, from which the uptake and efflux kinetics are reconstructed [17]. Various studies have provided important insight into transporter-mediated drug efflux and have suggested that drug accumulation may involve multiple kinetic components. However, while the measurements are derived from different cells at single time points, they do not capture uptake kinetics within the same individual cell over time. As a result, kinetic parameters obtained from population-level analyses represent averaged behavior that may obscure functionally relevant variability in drug uptake and retention among individual cells.

A critical consequence of population-level kinetic analysis is the inability to resolve cell-to-cell heterogeneity in drug uptake and retention. Even within clonal leukemia cell populations, individual cells can exhibit substantial variability in uptake rates, maximal intracellular drug accumulation, and responses to MDR modulation by inhibitors. As a result, comparisons between inhibitor-treated and untreated cell populations may yield ambiguous or misleading outcomes when variability across individual cells masks the true drug transport effects. Previous single-cell studies have shown the ability of drug-resistant cells without inhibitor treatment to display intracellular DNR retention comparable to, or even exceeding, that of inhibitor-treated cells during both uptake [18] and efflux [19] phases when evaluated across different individual cells. This functional heterogeneity complicates the assessment of MDR reversal and highlights the need for analytical approaches capable of isolating drug transport behavior at the level of individual cells [20].

To overcome the confounding effects of cell-to-cell heterogeneity, same-single-cell analysis (SASCA) was developed as an approach that enables direct comparison of drug transport behavior within the same individual cell under different experimental conditions [19,21]. This microfluidic method has been successfully applied to analyze single cancer cells obtained from blood samples of lung cancer patients [22,23]. By evaluating drug uptake or efflux before and after pharmacological modulation in the same individual cell, SASCA eliminates variability arising from the intercellular differences and enables observed changes in drug transport to be directly linked to the action of the applied inhibitor. Implementation of SASCA has been enabled by microfluidic biochip platforms that permit stable single-cell retention and continuous fluorescence monitoring, making it possible to perform real-time kinetic measurements over extended periods of time.

In this study, real-time DNR uptake was measured at the single-cell level in drug-sensitive (CEM/WT) and multidrug-resistant (CEM/VLB) leukemia cells retained within a microfluidic single-cell biochip. Continuous fluorescence monitoring enabled long-term observation of intracellular drug accumulation in individual cells, and kinetic modeling was applied to characterize uptake behavior under different resistance states. Analysis of the single-cell uptake data revealed substantial cell-to-cell variation in both the rate of DNR uptake and maximal intracellular drug retention in drug-sensitive and drug-resistant populations. This variability, which persisted following treatment with an MDR inhibitor, had obscured the apparent differences between inhibitor-treated and untreated cells when uptake was compared across different individual cells. The limitation of variability is overcome by directly comparing the drug uptakes in the same single cell before and after inhibitor exposure, thus allowing the MDR modulation effects due to inhibitors to be resolved independently of intercellular variability.

## 2. Materials and Methods

### 2.1. Microfluidic Biochip Design

**Figure 1:**
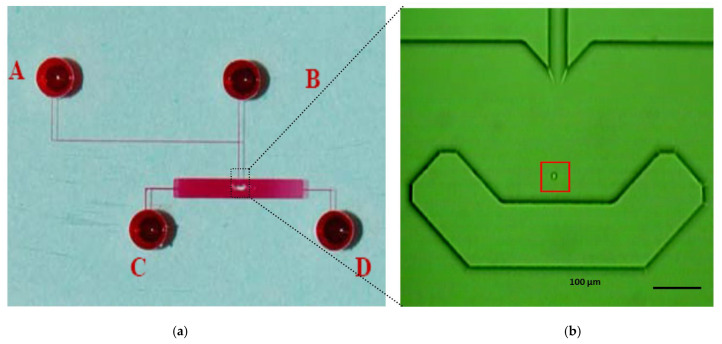
Microfluidic single-cell biochip used for real-time drug uptake measurements. (**a**) The image of the glass microfluidic biochip (filled with red-dyed solution) shows four fluidic channels connected to four reservoirs A, B, C, D (2 mm diameter) and a central observation chamber incorporating a U-shaped single-cell retention structure. (**b**) Optical micrograph of a single leukemia cell hydrodynamically trapped within the retention structure during continuous perfusion. The red box depicts the detection window of the single cell.

The microfluidic single-cell biochip used in this study is shown in Figure 1. The glass chip consisted of four fluidic channels connected to four reservoirs and a central observation chamber incorporating a U-shaped single-cell retention structure. This chip geometry enabled the stable hydrodynamic trapping of individual cells while permitting continuous perfusion of drug solutions [10,24,25]. Microfluidic isolation technologies have been widely applied for drug testing of single tumor cells and small cell clusters, enabling controlled exposure and real-time assessment of heterogeneous drug responses at the single-cell level [26]. The biochip was fabricated using standard glass microfabrication procedures by CMC Microsystems (Kingston, ON, Canada). The chip design that included a cell chamber and one cell retention structure for cell trapping was prepared by L-Edit Pro 8.3 (Tanner, Pasadena, CA, USA), and a GDSII file was generated. This was used to create the chrome photomask for UV exposure on the borofloat glass substrate coated with photoresist (SU-8). The standard photolithographic method was used to HF-etch the channels in the glass to a depth of 40 µm. The reservoirs were designed to accommodate sufficient solution volume for sustained experimental operation, with a depth of 600 μm and a diameter of 2 mm.

### 2.2. Reagents and Chemicals

Daunorubicin (DNR), vinblastine (VLB), cyclosporine A (CsA), and penicillin were obtained from Sigma-Aldrich (St. Louis, MO, USA). DNR was prepared as a stock solution prior to use, and CsA was used as a P-glycoprotein (P-gp) inhibitor in MDR modulation experiments. α-minimum essential medium (α-MEM) was purchased from Gibco (Grand Island, NY, USA), and fetal bovine serum (FBS) was obtained from the American Type Culture Collection (ATCC, Manassas, VA, USA). All reagents were of analytical grade, unless otherwise specified.

### 2.3. Cell Lines and Culture Conditions 

The CCRF-CEM human leukemia cell line, originally derived from a patient with acute lymphoblastic leukemia (ALL), was used in this study. Two related cell populations were examined, including the wild-type parental cell line (CEM/WT) and a MDR vinblastine-resistant subline (CEM/VLB1000). The CEM/VLB1000 cell line was generated through prolonged exposure to vinblastine (VLB), exhibiting a stable MDR phenotype associated with altered drug transport behavior mediated by ABC transporters [9,27]. Both CEM/WT and CEM/VLB1000 cells were cultured in a-MEM supplemented with 10% FBS and 50 U/mL penicillin. The cells were maintained at 37 °C in a humidified incubator containing 5% CO_2_. To preserve the cellular MDR phenotype, CEM/VLB1000 cells were continuously cultured in the presence of 1000 ng/mL vinblastine. Prior to drug uptake experiments, the cells were handled under identical culture conditions, and vinblastine was omitted during experimental measurements, unless otherwise specified.

### 2.4. Single-Cell Selection, Trapping, and Retention

**Figure 2:**
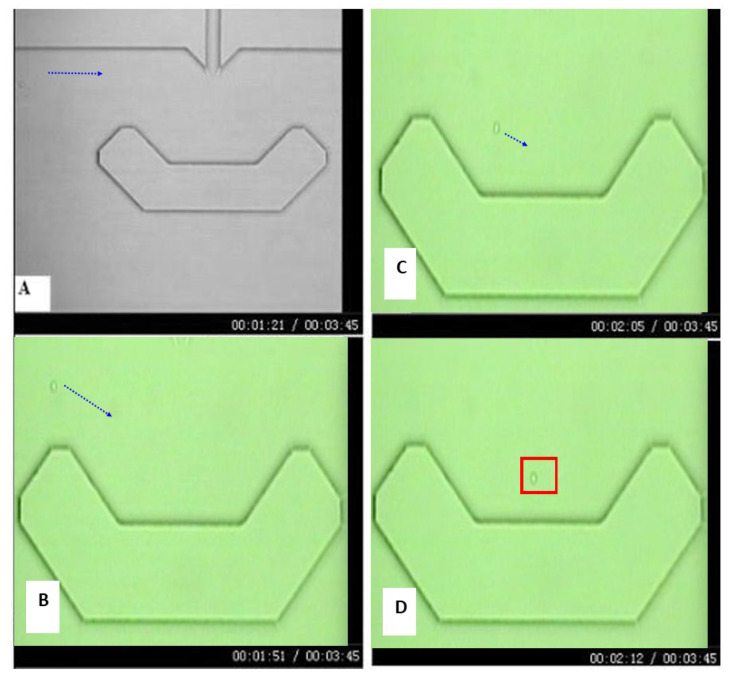
Time-lapse sequence of single-cell capture and retention within the microfluidic biochip. Sequential bright-field images showing a single leukemia cell entering the observation chamber, being guided toward the U-shaped retention structure, and becoming stably trapped for long-term measurement. The blue arrows represent the direction of the cell movement; the red box depicts the detection window for the single-cell measurement. The times of the capture images are (**A**) 0 s, (**B**) 30 s, (**C**) 44 s, (**D**) 51 s.

Single-cell experiments were conducted using the microfluidic biochip, as described in Figure 1. Cell suspensions were introduced into the microfluidic channels by gentle pressure-driven flow and directed toward the central observation chamber. The geometry of the chip enabled individual cells to be guided into a U-shaped retention structure, where hydrodynamic forces facilitated stable trapping of a single cell while allowing excess cells to pass through the channel. The process of single-cell capture and retention is illustrated by the time-lapse sequence shown in Figure 2, which depicts a representative CEM cell entering the observation chamber, being guided toward the retention structure, and becoming stably trapped. Flow conditions were adjusted to ensure that only one cell occupied the retention site at a time, minimizing cell displacement.

Following cell capture, the trapped cell was allowed to settle at the bottom of the observation chamber prior to fluorescence measurements. The entire cell selection and trapping process typically required approximately 2 min, after which an additional equilibration period of ~15 min was allowed to ensure stable positioning and cellular settling before initiation of real-time fluorescence acquisition. Continuous perfusion was maintained throughout the experiment, enabling long-term retention of the same individual cell and rapid exchange of extracellular solutions during drug uptake measurements and SASCA. The single-cell selection and retention procedure follows previously reported SASCA and hydrodynamic trapping approaches [19,24].

### 2.5. Real-Time Fluorescence Measurement and Data Acquisition

Real-time fluorescence measurements were performed to monitor intracellular accumulation of DNR in single retained cells. The microfluidic single-cell biochip was mounted on an inverted fluorescence microscope (Nikon TE300, Nikon Instruments, Konan, Minato-ku, Japan). A CCD camera was used to image cell morphology and verify stable single-cell retention, while changes in intracellular DNR fluorescence intensity were monitored using a photomultiplier tube (PMT) detector. The monitoring of the PMT signal was based on a measurement window defined by the red box in Figure 1b or Figure 2D. The intrinsic fluorescence of DNR served as the signal for quantifying intracellular drug accumulation [5,28].

Fluorescence signals within the detection window (Figure S1 in Supplementary Information) were acquired at fixed time intervals throughout each experiment to generate continuous time points of DNR uptake for individual cells. Cellular fluorescence intensity was quantified by subtracting the background fluorescence measured in a nearby cell-free region from the cellular signal. The resulting fluorescence intensity values were used as a measure of relative intracellular DNR accumulation over time. Another fluorescence image of a single cell is given in Figure S2.

DNR accumulation in drug-sensitive CEM/WT cells was measured in the absence of an inhibitor. For multidrug-resistant CEM/VLB1000 cells, fluorescence measurements were performed both in the absence and presence of the inhibitor CsA. In SASCA experiments, fluorescence time courses were recorded sequentially for the same individual cell before and after CsA introduction, enabling direct comparison of uptake kinetics under inhibited and non-inhibited conditions.

## 3. Results

### 3.1. Processing and Quantification of Real-Time Single-Cell DNR Fluorescence

**Figure 3:**
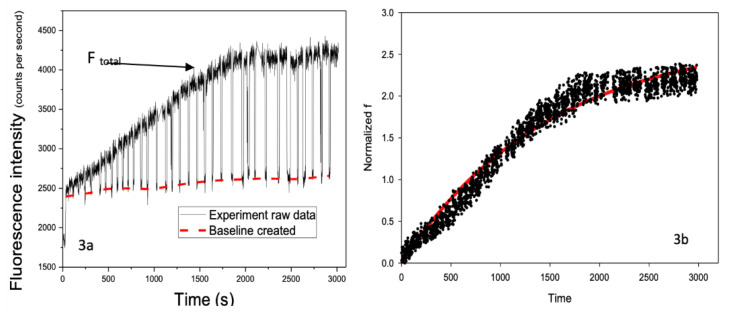
Real-time single-cell fluorescence signals during DNR uptake. (**a**) Representative raw fluorescence trace recorded from a single CEM cell (cell 148) during continuous perfusion with DNR, showing periodic peaks and valleys corresponding to total signal (F*_total_*) and background (baseline), respectively. (**b**) Background-subtracted and normalized intracellular fluorescence signal obtained after signal processing, used for subsequent kinetic analysis.

Following single-cell selection and retention within the microfluidic biochip, a DNR- containing solution was introduced to the cell under continuous perfusion. Real-time fluorescence signals arising from intracellular DNR accumulation were recorded for individual cells. A representative fluorescence trace measurement obtained from a single CEM cell is shown in Figure 3a.

The recorded signals exhibited peaks and valleys corresponding to the presence or absence, respectively, of the cell within the fluorescence detection window. The peak values represent the combined fluorescence signal from intracellular DNR and the surrounding extracellular environment, whereas the valleys represent background fluorescence originating from the extracellular solution alone. Using OriginLab, (Northampton, MA, USA) a baseline corresponding to background fluorescence (*F_background_*) was generated from the valley signal and subtracted from the total fluorescence signal (*F_total_*) to isolate the intracellular contribution (*F_cell_*), see Equation (1):

*F_cell_ = F_total_* − *F_background_* (1)

After background subtraction, transient spikes in the signal were removed, and the resulting intracellular fluorescence signal was normalized using the known intracellular DNR fluorescence intensity, see Figure 3b. This normalization enabled direct comparison of intracellular DNR accumulation across different cells without requiring removal of extracellular DNR, which is typically necessary in conventional flow cytometry-based assays.

### 3.2. DNR Uptake Kinetics in Drug-Sensitive Single Cells (CEM/WT)

**Figure 4:**
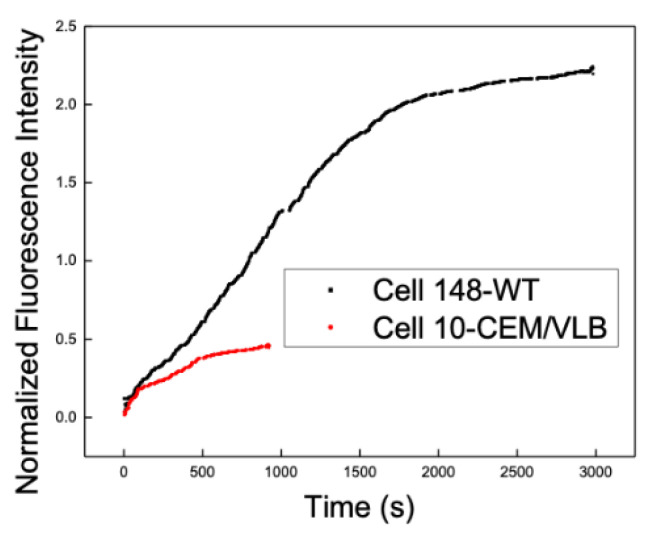
Real-time intracellular DNR uptake in representative drug-sensitive and MDR leukemia cells. Time-dependent normalized intracellular DNR fluorescence measured in a single CEM/WT cell and a single CEM/VLB cell under continuous perfusion, illustrating the differences in the uptake kinetics and maximum intracellular DNR accumulation between the two cell types.

To compare real-time DNR uptake behavior in drug-sensitive and MDR leukemia cells, representative intracellular DNR fluorescence traces were examined for CEM/WT and CEM/VLB cells. Figure 4 shows the overlaid, normalized time-dependent intracellular DNR fluorescence measured in a single CEM/WT cell and a single CEM/VLB cell.

In both cells, the intracellular DNR fluorescence increased rapidly during the initial phase of exposure, followed by a gradual decrease in the uptake rate to approach a plateau. The maximum intracellular DNR accumulation observed in the CEM/VLB cell was substantially lower than that observed in the CEM/WT cell. In addition, the time required for intracellular DNR fluorescence to reach approximately half of the plateau value differed between the two types of single cells, occurring at approximately 1100 s for the CEM/WT cell and approximately 400 s for the CEM/VLB cell.

During the initial uptake phase, intracellular DNR fluorescence increased approximately linearly within the first 100 s in both cell types. Among CEM/WT cells, the subsequent uptake behavior varied considerably between individual cells, with differences observed in both the rate at which the uptake slowed down and the level at which the intracellular DNR accumulation approached a plateau. This variability was evident across multiple CEM/WT cells and was reflected in the differences in overall uptake kinetics and maximal intracellular fluorescence levels, underscoring substantial cell-to-cell heterogeneity in DNR uptake even within the drug-sensitive cell population.

### 3.3. DNR Uptake Kinetics in Multidrug-Resistant Single Cells (CEM/VLB1000)

To quantitatively characterize DNR uptake kinetics in drug-sensitive leukemia cells, Sigma plot 11 was used for analysis, and the normalized intracellular fluorescence time courses obtained from CEM/WT cells were fitted using a one-exponential uptake model [18]:

*f* = *P* (1 − *e^−Qt^*) (2)

In Equation (2), *f* represents the normalized intracellular DNR fluorescence at time *t*, *P* is the pre-exponential factor corresponding to the asymptotic intracellular DNR accumulation, and *Q* is the uptake rate constant. 

**Figure 5:**
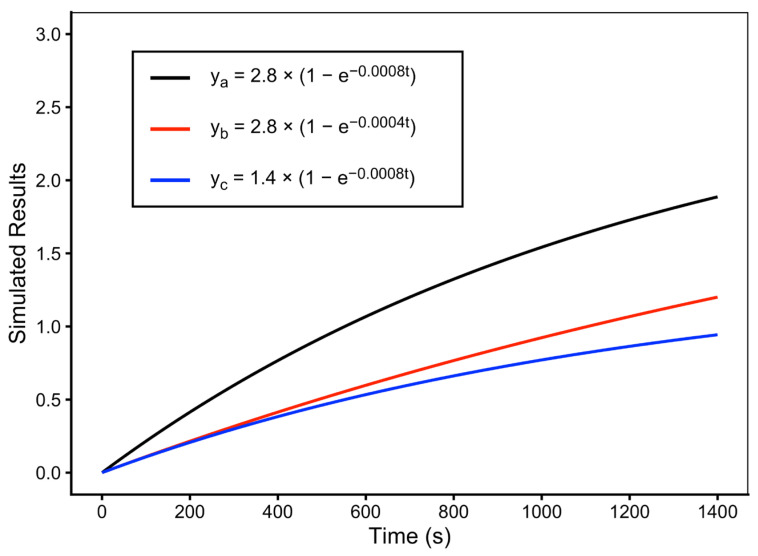
Simulated results with different *P* and *Q* values. The simulation, with y = normalized fluorescence intensity and x = time in seconds, was based on the one-exponential uptake model as given by Equation (2). Curve a was constructed using the fitted values of cell 148 obtained in Table 1.

Figure 3b shows the representative experimental DNR uptake curves from a CEM/WT cell (cell 148) together with the corresponding one-exponential fit, also depicted in Figure 4 (black curve) and Figure 5 (black curve). The fitted curve shown in Figure 5 was generated using the fitted kinetic parameters (for Equation (2)) obtained from cell 148, highlighted in Table 1, and was selected as a representative example due to its relatively high uptake rate and substantial intracellular DNR accumulation while maintaining good agreement between the experimental data and the fitted model. The one-exponential model closely followed the experimentally measured fluorescence trace (Figure 3b) over the full duration of the measurement, capturing both the rapid initial increase in intracellular DNR fluorescence and the subsequent gradual approach to a plateau. These results indicate that the one-exponential model provides an appropriate description of DNR uptake kinetics in drug-sensitive single cells.

In addition, Figure 5 illustrates simulated uptake curves generated using representative fitted values of *P* and *Q* obtained from individual CEM/WT cells. Variations in *Q* produced differences in the rate at which intracellular DNR fluorescence increased toward the plateau, whereas variations in *P* altered the asymptotic level of intracellular DNR accumulation. These simulations demonstrate the sensitivity of the uptake kinetics to changes in the fitted kinetic parameters.

Table 1 summarizes the fitted kinetic parameters obtained from one-exponential modeling of DNR uptake in five individual CEM/WT cells. The correlation coefficient (R) values indicate good agreement between the experimental data and the one-exponential model across all cells analyzed. Substantial cell-to-cell variability was observed in both fitted parameters (P and Q). The uptake rate constant *Q* ranged from 0.0002 to 0.0008 s^−1^, corresponding to a fourfold difference in uptake rate among individual cells. Similarly, the pre-exponential factor *P*, representing the maximum normalized intracellular DNR accumulation, varied by approximately 1.8-fold across the cells analyzed. These results demonstrate the pronounced heterogeneity in DNR uptake kinetics among drug-sensitive leukemia cells, even under identical experimental conditions.

To further evaluate whether additional uptake processes contributed to DNR accumulation, a two-exponential uptake model [26] was applied to the normalized intracellular fluorescence time measurements:

*f = A*(1 *− e^−Bt^*) *+ C*(1 *− e^−Dt^*) (3)

In the equation, *f*(*t*) represents the normalized intracellular DNR fluorescence at time *t*. parameters *A* and *C* correspond to the asymptotic contributions of the two uptake components, while *B* and *D* represent the corresponding uptake rate constants.

The two-exponential model was first applied to DNR uptake data obtained from CEM/WT cells. As summarized in Table 1, both the one-exponential and two-exponential models yielded similar correlation coefficient (R) values.

**Figure 6:**
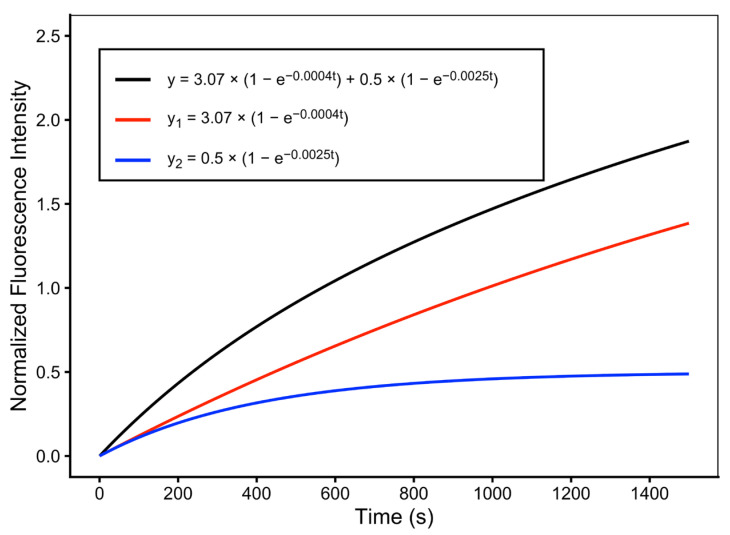
Two-exponential fitting of DNR uptake in a drug-sensitive leukemia cell (CEM/WT). Normalized intracellular DNR fluorescence time course for CEM/WT cell 148 fitted using the two-exponential uptake model. Curve 1 represents the first exponential uptake component, curve 2 represents the second exponential component, and curve 3 represents the overall two-exponential fit. The second exponential component contributes minimally to the overall uptake profile.

However, comparison of the fitted parameters revealed that the second exponential term contributed minimally to the overall uptake behavior in CEM/WT cells. In particular, the fitted values of *C* were substantially smaller than the corresponding values of *A*. This is illustrated in Figure 6, which shows the two-exponential fit for cell 148, where the second exponential component (curve y2) is negligible relative to the primary component (curve y1).

Statistical comparison of the one-exponential and two-exponential models using an F-test further indicated that inclusion of the second exponential term did not significantly improve the fit for CEM/WT cells. These results support the use of the one-exponential model to describe DNR uptake in drug-sensitive leukemia cells.

### 3.4. Cell-to-Cell Variability in MDR Cells

In contrast, the application of the two-exponential model to DNR uptake data obtained from CEM/VLB cells revealed distinct behavior. In these cells, the fitted values of *A* and *C* were comparable in magnitude and indicated that both exponential terms contributed substantially to the overall uptake profile. As a result, the second exponential term cannot be neglected.

**Figure 7:**
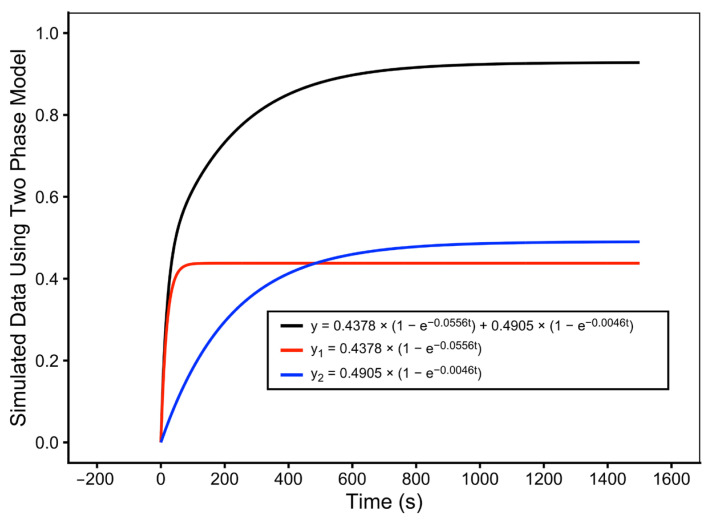
Two-exponential fitting of DNR uptake in a multidrug-resistant leukemia cell (CEM/VLB). Normalized intracellular DNR fluorescence time course for a single CEM/VLB cell (cell 58) fitted using the two-exponential uptake model. Curve 1 and curve 2 represent the two exponential uptake components, and the top curve represents the overall two-exponential fit.

Statistical comparison using the F-test demonstrated that the two-exponential model provided a better fit for the experimental data for CEM/VLB cells than the one-exponential model. Figure 7 shows a representative two-exponential fit for DNR uptake in a single CEM/VLB cell (cell 58), illustrating the contribution of both exponential components to the overall drug uptake behavior.

The two-exponential uptake model was next applied to DNR accumulation measured in twenty individual CEM/VLB cells. The fitted kinetic parameters obtained from this analysis are summarized in Table 2. In contrast to drug-sensitive cells, both exponential components contributed substantially to the overall uptake behavior in MDR cells.

Across the analyzed CEM/VLB cells, the fitted parameter *A* ranged from 0.0768 to 0.7980, while *C* ranged from 0.1147 to 0.700. Therefore, the maximum DNR retention, expressed as A+C, varied widely from 0.3862 to 1.4980 among individual cells. The uptake rate constants, B and D, also varied considerably across cells, indicating pronounced heterogeneity in both the magnitude and rate of DNR uptake in the MDR cell population. 

This substantial cell-to-cell variability in DNR uptake kinetics has been qualitatively reported previously, which has thus motivated the development of the SASCA approach [15]. In the present study, this variability is quantitatively captured for the first time using mathematical modeling of real-time single-cell uptake data. 

### 3.5. Comparison of DNR Uptake in MDR Cells with and Without CsA Across Different Cells

To evaluate the effect of P-glycoprotein inhibition on DNR uptake at the single-cell level, two-exponential kinetic modeling was also performed for DNR accumulation in CEM/VLB cells in the presence of cyclosporine A (CsA). The fitted parameters obtained from these experiments are summarized in Table 3.

When the results obtained in the presence of CsA (Table 3) were compared with those obtained in the absence of CsA (Table 2), no clear enhancement of intracellular DNR retention could be resolved across different cells. The fitted values of A, C, and A + C overlapped substantially between the two groups, indicating that cell-to-cell variability dominated the observed uptake behavior.

**Figure 8:**
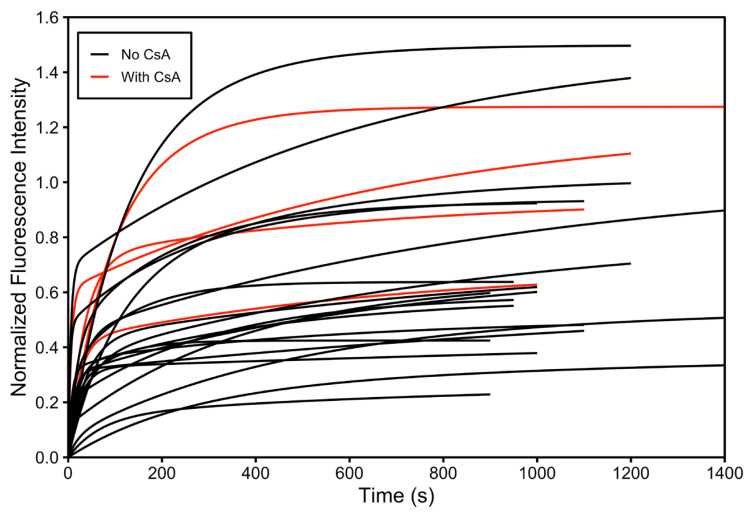
Comparison of DNR uptake in multidrug-resistant leukemia cells with and without CsA across different cells. Normalized intracellular DNR fluorescence time courses measured in individual CEM/VLB cells in the presence (red) and absence (black) of cyclosporine A. The twenty black uptake curves represent 20 individual single cells measured in the absence of CsA, whereas the four red curves represent 4 individual single cells measured in the presence of CsA. These curves from single cells under the two conditions overlap, indicating substantial cell-to-cell variability.

Figure 8 shows representative DNR uptake curves obtained from CEM/VLB cells measured with and without CsA treatment. The uptake profiles from the two conditions were interspersed, and no distinct clustering of curves corresponding to inhibitor-treated versus untreated cells was observed. Statistical comparison using the F test further confirmed that no significant difference could be resolved between the two groups when uptake was compared across different single cells. There is no indication that the average of the four red curves (with CsA) is higher than that of the 20 black curves (no CsA). This negative conclusion has been observed previously in 10 cells [11].

Taken together, these results demonstrate that comparison of DNR uptake across different single MDR cells, even when kinetic modeling is applied, does not allow reliable resolution of MDR inhibition effects due to substantial intrinsic cell-to-cell variability. Under these conditions, no definitive conclusion regarding enhanced intracellular DNR retention following CsA treatment can be drawn using cross-cell analysis alone, consistent with limitations previously reported for different-single-cell analysis (DISCA) approaches [19].

### 3.6. SASCA Method Used to Overcome Cellular Variations in Drug Uptake

To overcome this limitation, a same-single-cell analysis (SASCA) approach was employed, in which DNR uptake was measured sequentially in the same individual cell before and after MDR inhibitor exposure. By directly comparing uptake behavior within the same cell under different conditions, this approach eliminates intercellular variability and enables resolution of drug modulation effects that are obscured in cross-cell analyses.

**Figure 9:**
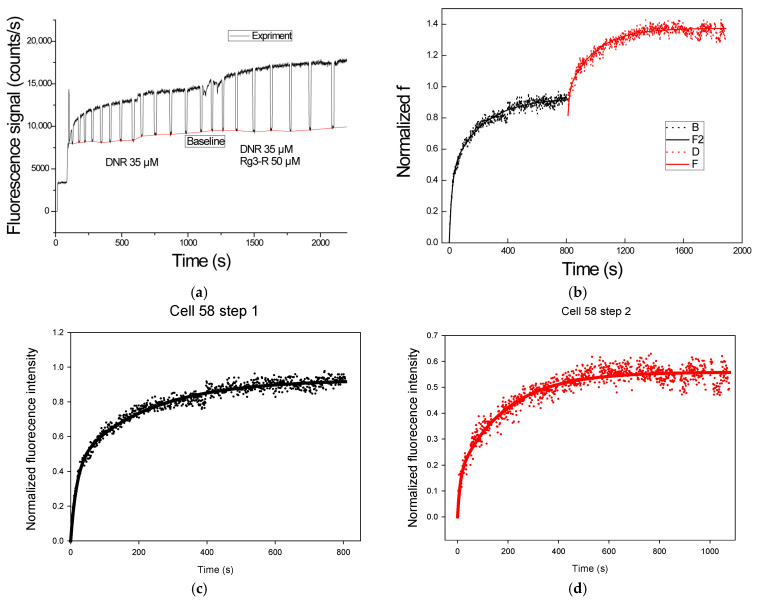
Same-single-cell analysis (SASCA) of DNR uptake in an MDR leukemia cell. (**a**) Raw fluorescence signal acquired during sequential DNR uptake measurements in a single CEM/VLB cell (cell 58). (**b**) Background-subtracted and normalized intracellular DNR fluorescence trace showing two uptake phases corresponding to control and inhibitor-treated conditions. (**c**) Two-exponential curve fitting for the control uptake phase. (**d**) Two-exponential curve fitting for the inhibitor-treated uptake phase.

The same-single-cell analysis (SASCA) method is used in order to rule out the cell-to-cell variation. Figure 9 shows the measurement of DNR uptake inside a single CEM/VLB cell. DNR was first introduced to the cell, and after the DNR uptake had reached a steady state at 1000s, the solution was changed to a mixture of DNR in the presence of an MDR inhibitor. As expected, the intracellular DNR started to increase again until it reached a second steady state. It is thus clear that there is an inhibition effect on the cell, and we can compare the 2 steps in terms of the A and C values.

Figure 9 shows a representative SASCA experiment performed on a single CEM/VLB cell. DNR was first introduced to the cell, and intracellular fluorescence was monitored until a steady-state level was reached at approximately 1000 s. The perfusion solution was then switched to a mixture of DNR and an MDR inhibitor, after which intracellular DNR fluorescence increased further before reaching a second steady state. Figure 9a shows the raw fluorescence signal acquired during the experiment. After background subtraction and normalization, the processed intracellular DNR fluorescence trace is shown in Figure 9b. Figure 9c,d show the corresponding two-exponential fits for the first (control) and second (inhibitor-treated) uptake phases, respectively. The fitted curves closely followed the experimentally measured data in both phases, with correlation coefficients (R) of 0.9867 for the control step and 0.9610 for the inhibitor-treated step, enabling quantitative comparison of uptake kinetics within the same cell.

Table 4 summarizes the fitted two-exponential kinetic parameters obtained from SASCA experiments performed on individual CEM/VLB cells, including cell 58, under the control conditions and in the presence of MDR inhibitor treatment. For cell 58, the uptake rate constant *B* increased following inhibitor exposure, corresponding to an approximately 1.8-fold increase relative to the DNR control condition (0.0973 vs. 0.0556). In addition, the combined asymptotic uptake parameter (*A + C*) increased following inhibitor treatment, corresponding to an approximately 1.4-fold increase in maximum normalized intracellular DNR accumulation compared to the control condition.

Similar trends were observed in additional CEM/VLB cells analyzed using the SASCA approach, with consistent increases in uptake rate and intracellular DNR accumulation following inhibitor treatment when comparisons were performed within the same individual cell.

Taken together, these results indicate that same-single-cell analysis enables quantitative resolution of changes in DNR uptake kinetics and intracellular retention following MDR inhibitor treatment, which could not be reliably detected using cross-cell comparisons due to intrinsic cell-to-cell variability.

## 4. Discussion

This study demonstrates that real-time measurement of daunorubicin uptake at the single-cell level provides quantitative insight into MDR cell dynamics that cannot be resolved using population-averaged approaches. Others used droplet-based microfluidics to study single-cell dynamics [29,30,31]. Investigations of cytotoxicity, cytokine capture, and exosome secretion of single cells were also achieved using droplet microfluidics [32,33,34,35]. In our hands, using a microfluidic single-cell biochip, we observed pronounced heterogeneity in daunorubicin uptake kinetics among both drug-sensitive and multidrug-resistant leukemia cells, even under identical experimental conditions. Such variability directly affects intracellular drug accumulation and has important implications for therapeutic response in leukemia, where MDR remains a major pathophysiological barrier to effective anthracycline-based chemotherapy [1,2,9], a limitation highlighted in recent single-cell analyses of MDR [13]. These findings reinforce the notion that intracellular drug exposure is governed through transporter expression in addition to cell-specific transport behavior that cannot be captured by bulk measurements [5,15].

Quantitative kinetic modeling revealed that daunorubicin uptake in drug-sensitive CEM/WT cells was well described by a single dominant uptake process, whereas multidrug-resistant CEM/VLB cells required a two-exponential model, indicating a more complex and kinetically distinct transport pathway [20,22]. Importantly, we further show that intrinsic cell-to-cell variability can obscure the effects of MDR inhibition when uptake behavior is compared across different cells, and that this limitation can be overcome using SASCA [19,24]. The requirement for a two-exponential model in MDR cells suggests the presence of at least two kinetically distinct uptake components that are absent or negligible in drug-sensitive cells. While this study does not assign specific biological mechanisms to individual exponential terms, the emergence of additional kinetic components in MDR cells is consistent with previous observations of altered drug transport kinetics in resistance cancer cell populations [2,17,27]. These findings highlight that MDR is associated with altered single-cell uptake kinetics, emphasizing the importance of resolving temporal drug transport behavior when evaluating intracellular drug accumulation in resistant leukemia cells.

A central outcome of this work is the demonstration that intrinsic cell-to-cell variability can obscure the effects of MDR inhibition when uptake behavior is compared across different single cells. Although kinetic modeling was able to describe uptake behavior within individual cells, comparisons across cells did not consistently reveal enhanced daunorubicin retention following inhibitor treatment, as the fitted parameters from inhibited and non-inhibited cells substantially overlapped. This limitation is analogous to what was encountered in different-single-cell analysis approaches, where variability between cells masked true modulation effects [19]. In contrast, SASCA enabled direct comparison of uptake kinetics within the same individual cell before and after inhibitor exposure, thereby removing intercellular variability from the analysis and allowing inhibitor-induced changes in drug uptake and retention to be clearly resolved [19,24,36]. This within-cell comparison framework provides a more sensitive and reliable means of evaluating MDR modulation at the single-cell level and supports the broader application of longitudinal single-cell measurements for studying drug transport behavior in heterogeneous leukemia cell populations [10,21,24].

## 5. Conclusions

In this study, we employed a microfluidic single-cell biochip combined with real-time fluorescence monitoring and kinetic modeling to investigate DNR uptake in drug-sensitive and MDR leukemia cells. This approach enabled continuous measurement of intracellular drug accumulation in individual cells, overcoming the limitations of population-averaged techniques that obscure dynamic transport behavior and intrinsic cellular heterogeneity.

Our kinetic analysis revealed distinct uptake mechanisms in the two cell populations. DNR accumulation in drug-sensitive CEM/WT cells was well described by a single-exponential model, consistent with a dominant uptake process. In contrast, uptake in MDR CEM/VLB cells required a two-exponential model, indicating the presence of two kinetically distinct processes, likely reflecting altered transport kinetics associated with P-glycoprotein activity and intracellular drug handling. Substantial intercellular variability was observed in both uptake rates and maximal intracellular drug retention in all cell populations. This heterogeneity significantly limited the ability to assess MDR reversal when uptake data were compared across different individual cells, even in the presence of an MDR inhibitor. To address this challenge, we applied SASCA, enabling direct comparison of drug uptake before and after inhibitor treatment. This approach eliminated intercellular variability and revealed enhanced intracellular DNR retention and accelerated uptake kinetics following inhibition.

In summary, these results demonstrate that real-time single-cell kinetic analysis combined with same-cell comparison provides a robust framework for resolving functionally relevant heterogeneity in drug-resistant cancer cells. This platform offers mechanistic insight into MDR that cannot be obtained from population-level measurements and may improve the evaluation drug transport, resistance mechanisms, and therapeutic modulation in limited or heterogeneous cell samples. Future investigations may also benefit from integrating insights from P-gp biology and emerging nanomedicine-based drug delivery systems designed to counteract efflux and enhance intracellular retention of chemotherapeutic agents.

## Figures and Tables

**Table 1:** One-exponential and two-exponential fittings for DNR uptake in individual CEM/WT cells. For the one-exponential fitting (Equation (2)), the parameters are P and Q, and for the two-exponential fitting (Equation (3)), the fitting parameters are A–D. R is the correlation coefficient. Kinetic parameters are obtained from one-exponential and two-exponential fittings of the normalized intracellular DNR fluorescence time courses. Cell 148 (highlighted in red) was selected as a representative example for kinetic modeling shown in Figure 5.

		One-Exponent Fitting Summary			Two-Exponent Fitting Summary				
**Cell No.**	**Cell type**	***P***	***Q***	**R**	***A***	***B***	***C***	***D***	**R**
146	CEM/WT	2.7140	0.0007	0.9812	2.7000	0.0007	0.1000	0.0002	0.9778
147	CEM/WT	3.7468	0.0002	0.9241	2.8000	0.0002	0.3000	0.0003	0.9230
148	CEM/WT	2.8010	0.0008	0.9715	3.0700	0.0004	0.5000	0.0025	0.9712
149	CEM/WT	2.8184	0.0004	0.9685	3.0376	0.0003	0.3150	0.0006	0.9665
154	CEM/WT	2.3556	0.0007	0.949	3.3500	0.0003	0.3500	0.0006	0.9306

**Table 2:** Two-exponential fitting parameters for DNR uptake in individual CEM/VLB cells. Parameters obtained from two-exponential modeling of normalized intracellular DNR fluorescence time courses measured in twenty single multidrug-resistant leukemia cells (CEM/VLB).

Cell No.	Cell Type	A	B	C	D	R	A + C
10	CEM/VLB	0.0768	0.0442	0.4581	0.0020	0.9757	0.5349
11	CEM/VLB	0.4455	0.0400	0.6000	0.0010	0.9520	1.0455
24	CEM/VLB	0.7980	0.0100	0.7000	0.0051	0.9306	1.4980
56	CEM/VLB	0.3272	0.0405	0.2000	0.0003	0.8597	0.5272
58	CEM/VLB	0.4378	0.0556	0.4905	0.0046	0.9865	0.9283
69	CEM/VLB	0.3495	0.0517	0.1563	0.0017	0.8774	0.5058
70-01	CEM/VLB	0.1495	0.0143	0.1147	0.0013	0.7012	0.2642
70-03	CEM/VLB	0.2011	0.1483	0.3897	0.0032	0.8513	0.5908
70-07	CEM/VLB	0.1150	0.2720	0.5339	0.0026	0.8878	0.6489
70-08	CEM/VLB	0.3540	0.0264	0.4782	0.0011	0.8894	0.8322
70-11	CEM/VLB	0.3000	0.0864	0.4000	0.0014	0.8551	0.7000
70-12	CEM/VLB	0.3145	0.0815	0.3005	0.0006	0.7385	0.6150
70-18	CEM/VLB	0.4303	0.0269	0.3182	0.0009	0.8770	0.7485
70-19	CEM/VLB	0.3191	0.1375	0.2646	0.0022	0.7717	0.5837
70-23	CEM/VLB	0.6220	0.0100	0.4000	0.0023	0.9095	1.0220
70-25	CEM/VLB	0.6922	0.1792	0.8048	0.0016	0.8822	1.4970
76	CEM/VLB	0.1975	0.0835	0.2269	0.0134	0.7056	0.4244
77	CEM/VLB	0.3040	0.0894	0.3337	0.0081	0.7817	0.6377
88	CEM/VLB	0.4827	0.2513	0.4565	0.0037	0.8593	0.9392
95	CEM/VLB	0.2510	0.0040	0.1352	0.0007	0.9140	0.3862

**Table 3:** Two-exponential fitting parameters for DNR uptake in individual CEM/VLB cells in the presence of CsA. Parameters obtained from two-exponential modeling of normalized intracellular DNR fluorescence time courses measured in single multidrug-resistant leukemia cells treated with cyclosporine A.

	Cell No.	Cell Type	A	B	C	D	R	A + B
13-07-16	150	CEM/VLB CsA	0.6109	0.1526	0.6066	0.0014	0.8682	1.2175
13-07-17-1	151	CEM/VLB CsA	0.5281	0.015	0.7463	0.007	0.8911	1.2744
13-07-17-2	152	CEM/VLB CsA	0.7277	0.0293	0.2146	0.0015	0.774	0.9423
13-07-17-3	153	CEM/VLB CsA	0.4251	0.0446	0.2692	0.0014	0.773	0.6943

**Table 4:** Two-exponential fitting parameters for DNR uptake measured using same-single-cell analysis (SASCA). Fitted kinetic parameters obtained from two-exponential modeling of normalized intracellular DNR fluorescence measured sequentially in individual CEM/VLB cells under control conditions and following MDR inhibitor treatment.

	Step	A Value	B Value	C Value	D Value
Cell 58	Same-cell Control	0.4378	0.0556	0.4905	0.0046
	With Inhibitor Rg3-R 50 µM	0.1614	0.0973	0.3972	0.0053
Cell 70-08	Same-cell Control	0.3540	0.0264	0.4782	0.0011
	With Inhibitor Rg3-R 50 µM	0.2055	0.0371	0.2454	0.0060
Cell 95	Same-cell Control	0.2510	0.0040	0.1352	0.0007
	With Inhibitor Rg3-R 50 µM	0.2681	0.0050	0.1000	0.0080

## Data Availability

The original contributions presented in this study are included in the article/Supplementary Material. Further inquiries can be directed to the corresponding author.

## Supplementary Materials

The following supporting information can be downloaded at: https://www.mdpi.com/article/10.3390/pathophysiology33020028/s1, Figure S1: Green fluorescence images of a yeast cell due to fluorescein as excited by blue light (a) when the cell was measured inside the detection window and (b) when the solution background beside the cell was measured; Figure S2: Image of an ovarian cell (NCI/ADR-RES) retained in the cell retention structure (a) blue at the beginning of the experiment due to the blue light, (b) orange fluorescence due to DNR during experiment of DNR accumulation, and (c) after the experiment.

## Abbreviations

The following abbreviations are used in this manuscript:

ALL	Acute Lymphoblastic Leukemia
CsA	Cyclosporin A
DNR	Daunorubicin
DISCA	Different-Single-Cell Analysis
FBS	Fetal Bovine Serum
MDR	Multidrug Resistance
MDR1	Multidrug Resistance Protein 1
PBS	Phosphate-Buffered Saline
P-gp	P-Glycoprotein
PMT	Photomultiplier Tube
SASCA	Same-Single-Cell Analysis
VLB	Vinblastine
WT	Wild Type
