# Supplementary material for: Real-Time Single-Cell Measurement and Kinetic Modeling of Daunorubicin Uptake in Multidrug-Resistant Leukemia Cells Using a Microfluidic Biochip
Source: Pathophysiology. 2026 Apr 21;33(2):28. doi: 10.3390/pathophysiology33020028 (PMC13108144; doi:10.3390/pathophysiology33020028)
Supplement: Supplementary file 1 [file pathophysiology-33-00028-s001.zip › pathophysiology-4134911-supplementary.pdf]

Supplementary Information

Real-Time Single-Cell Measurement and Kinetic Modeling of Daunorubicin Uptake in Multidrug-Resistant Leukemia Cells Using a Microfluidic Biochip

Yuchun Chen, Megan Chiem, Nandini Joshi, Paul C. H. Li*

Department of Chemistry, Simon Fraser University, Burnaby, BC V5A 1S6, Canada *Correspondence: paulli@sfu.ca

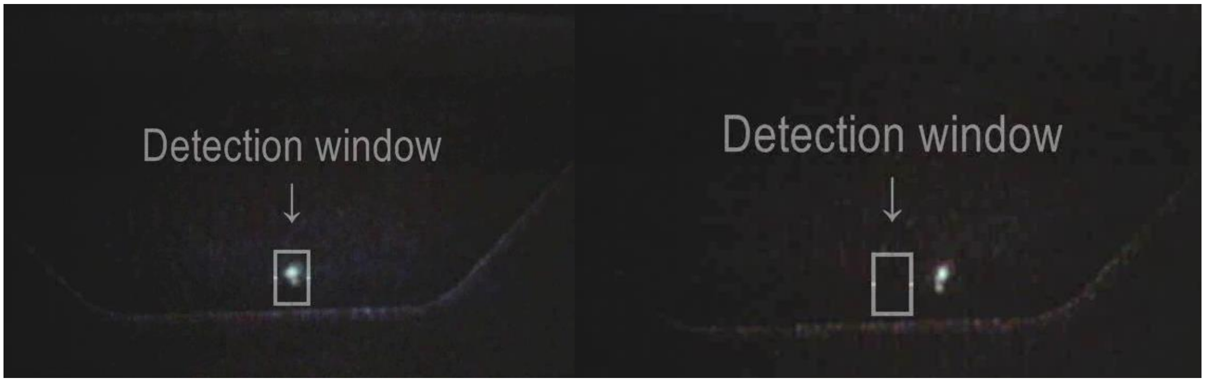

(**a**) (**b**)

Figure S1: Green fluorescence images of a yeast cell due to fluorescein as excited by blue light (a) when the cell was measured inside the detection window and (b) when the solution background beside the cell was measured

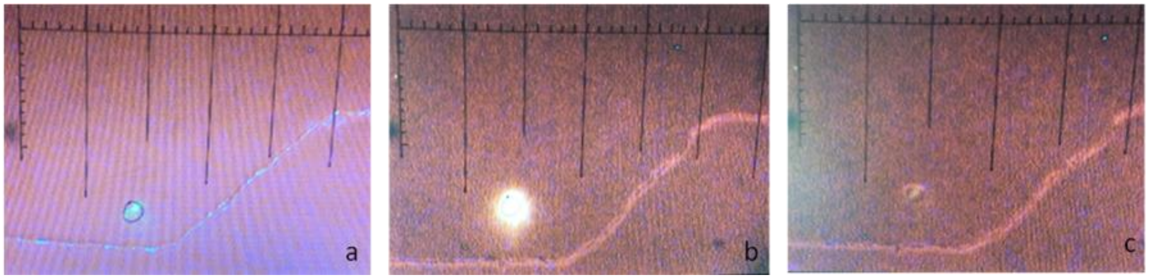

Figure S2: Image of an ovarian cell (NCI/ADR-RES) retained in the cell retention structure (a) blue at the beginning of the experiment due to the blue light, (b) orange fluorescence due to DNR during experiment of DNR accumulation, and (c) after the experiment.
